# Noninvasive detection of lung cancer using exhaled breath

**DOI:** 10.1002/cam4.162

**Published:** 2013-11-20

**Authors:** Xiao-An Fu, Mingxiao Li, Ralph J Knipp, Michael H Nantz, Michael Bousamra

**Affiliations:** 1 Department of Chemical Engineering, University of LouisvilleLouisville, Kentucky, 40208; 2 Department of Chemistry, University of LouisvilleLouisville, Kentucky, 40208; 3 Department of Surgery, University of LouisvilleLouisville, Kentucky, 40208; 4 James Graham Brown Cancer Center, University of LouisvilleLouisville, Kentucky, 40208

**Keywords:** Biomarkers, breath analysis, early diagnosis of cancer, lung cancer, volatile organic compounds

## Abstract

Early detection of lung cancer is a key factor for increasing the survival rates of lung cancer patients. The analysis of exhaled breath is promising as a noninvasive diagnostic tool for diagnosis of lung cancer. We demonstrate the quantitative analysis of carbonyl volatile organic compounds (VOCs) and identification of lung cancer VOC markers in exhaled breath using unique silicon microreactor technology. The microreactor consists of thousands of micropillars coated with an ammonium aminooxy salt for capture of carbonyl VOCs in exhaled breath by means of oximation reactions. Captured aminooxy-VOC adducts are analyzed by nanoelectrospray Fourier transform-ion cyclotron resonance (FT-ICR) mass spectrometry (MS). The concentrations of 2-butanone, 2-hydroxyacetaldehyde, 3-hydroxy-2-butanone, and 4-hydroxyhexenal (4-HHE) in the exhaled breath of lung cancer patients (*n* = 97) were significantly higher than in the exhaled breath of healthy smoker and nonsmoker controls (*n* = 88) and patients with benign pulmonary nodules (*n* = 32). The concentration of 2-butanone in exhaled breath of patients (*n* = 51) with stages II though IV non–small cell lung cancer (NSCLC) was significantly higher than in exhaled breath of patients with stage I (*n* = 34). The carbonyl VOC profile in exhaled breath determined using this new silicon microreactor technology provides for the noninvasive detection of lung cancer.

## Introduction

The US National Lung Screening Trial recently found that periodic computed tomography (CT) screening of heavy smokers could reduce mortalities of lung cancer patients by as much as 20% 1,2. Currently, CT and bronchoscopy are the principal techniques used for lung cancer detection 3,4. In recent years, the analysis of exhaled breath has become an international research frontier because of its applicability for noninvasive diagnosis of diseases 5–17. Several approaches have been developed to analyze exhaled breath including the use of sensor arrays 7–9,15–17, proton-transfer reaction mass spectrometry (PTR-MS) 18–20, selected ion flow tube mass spectrometry (SIFT-MS) 21,22, and gas chromatography–mass spectrometry (GC–MS) 13,18,23–26. Real-time analysis of volatile organic compounds (VOCs) in exhaled breath by PTR-MS has been established to monitor variation in VOCs with time 27–31. Although some VOCs in exhaled breath have been reported as lung cancer markers, there has been no clinical adoption of breath analysis methods for diagnosis because of the large number of VOCs in exhaled breath and the lack of cancer-specific VOC markers for reliably predicting lung cancer 5–17.

Here, we describe the quantitative analysis of carbonyl VOCs in exhaled breath and the identification of specific carbonyl VOCs related to lung cancer stages and histology using silicon microreactors for the capture of carbonyl VOCs. Our approach only requires a patient to fill a 1-L Tedlar bag with exhaled breath. The breath sample can then be analyzed offsite by mass spectrometry.

Lung cancer causes oxidative stress and induces oxidase enzymes, in turn producing higher concentrations of specific VOCs in exhaled breath 5,32,33. Carbonyl VOCs are produced in biochemical pathways as intermediates, and some can be unique to a given pathway, such as lipid oxidation induced by free radicals 33. Therefore, we have focused on identification of carbonyl VOC markers of lung cancer in exhaled breath using the silicon microreactor technology that we developed for chemoselective capture and analysis of trace carbonyl VOC in air and exhaled breath.

## Material and Methods

### The silicon microreactors

**Figure 1:**
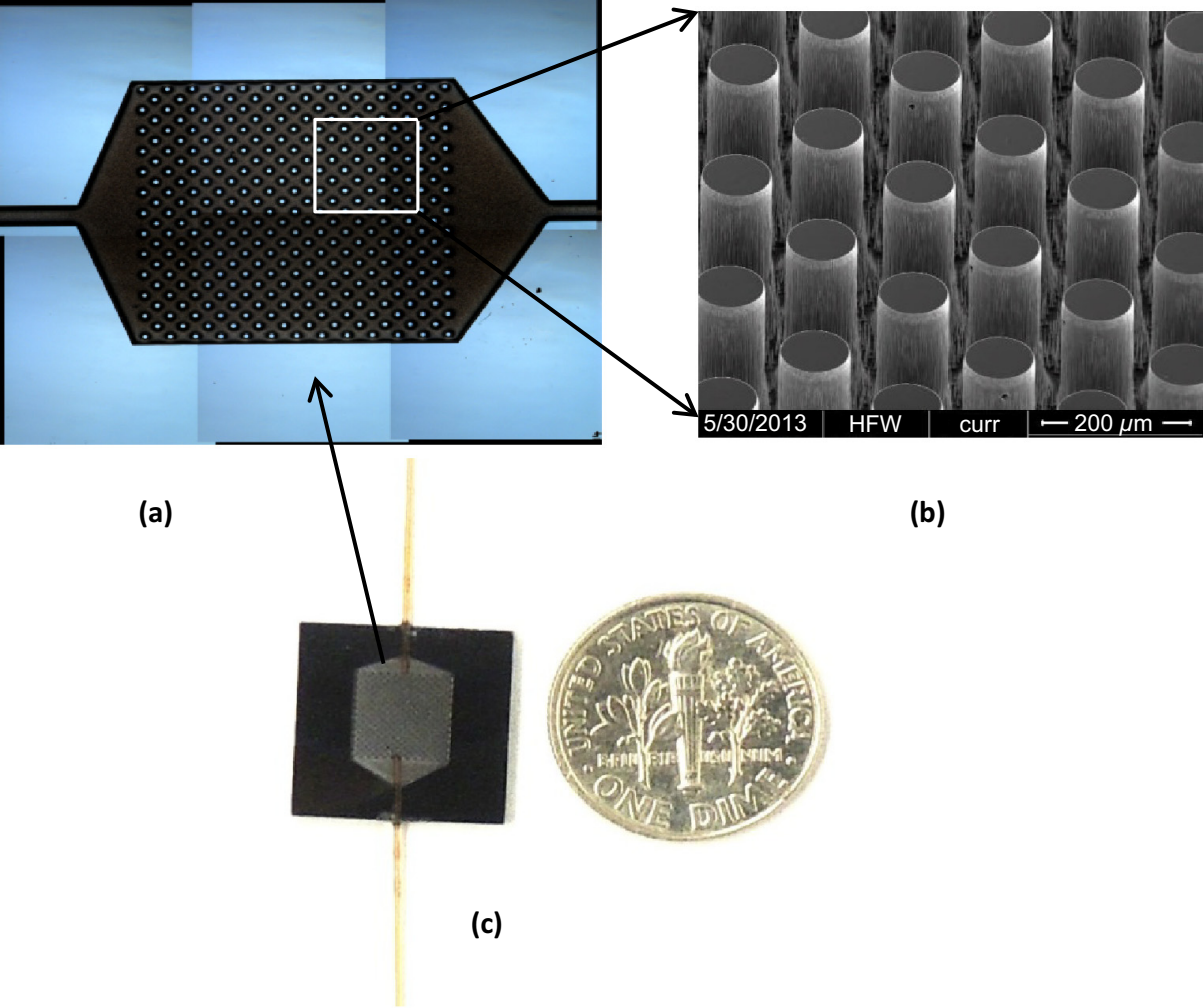
Capture of carbonyl volatile organic compounds (VOCs) in exhaled breath using a silicon microreactor. (a) The microreactor connected to two fused silica tubes. A dime was placed near the microreactor to indicate the size of the microreactor. (b) Optical micrograph of the microreactor before bonded with a glass wafer. (c) SEM micrograph of the micropillar array in the microreactor.

The silicon microreactors were fabricated from 4″-silicon wafers using standard microelectromechanical systems (MEMS) fabrication techniques. The detailed fabrication process has been published elsewhere 34,35. The microreactor shown in Figure 1(a) has size similar to a dime and consists of an array of micropillars defining a microfluidic channel (Fig. 1b). The micropillars have a high aspect ratio with a diameter of 50 *μ*m and height of 250 *μ*m (Fig. 1c) created by dry reactive ion etching (DRIE). The distance from center to center of the micropillars is 100 *μ*m. The channel size is 7 × 5 mm, with a total volume of about 5 *μ*L in the microreactor. The microreactor consists of over 5000 micropillars corresponding to a total micropillar surface area of about 260 mm^2^. The inlet and outlet of the microreactor were fitted with 190 *μ*m O.D. and 100 *μ*m I.D. deactivated fused silica tubes using a silica-based bonding agent (Fig. 1a).

The surface functionalization of the channels and micropillars with 2-(aminooxy)-*N*,*N*,*N*-trimethylethanammonium (ATM) iodide was performed by injecting ATM iodide in methanol solution of known concentration into the microreactor from one connection port followed by evaporation of the solvent under vacuum 34,35. The slightly negative surface charge of the silicon oxide micropillars allows for electrostatic binding of the cationic ATM on the surfaces of the micropillars. ATM reacts chemoselectively with trace carbonyl VOCs in exhaled breath by means of oximation with high reactivity.

### Exhaled breath specimen collection and processing

Air and exhaled breath samples were collected in 1-L Tedlar bags (Sigma-Aldrich, St. Louis, MO). The detailed research protocol for collection of exhaled breath samples was approved by the Institutional Review Board (IRB) at the University of Louisville. For the collection of exhaled breath samples, subjects would directly breathe into Tedlar bags through the Teflon tube, thus providing a noninvasive collection technique that was readily accepted by the patients. A 1-L breath sample was collected from a single exhaled breath; thus, a mixture of tidal and alveolar breath was collected. In this study, the CO_2_-controlled method of sampling exhaled breath was not performed. The advantage of the CO_2_-controlled method is to collect only alveolar end breath 36.

After collection of exhaled breath, the Tedlar bags were connected to the inlet port of the microreactor through one fused silica tube. The exit port of the microreactor was connected to a vacuum pump through the other fused silica tube on the microreactor as shown in Figure 1(a). The setup for capture of carbonyl VOCs includes a vacuum pump to pull gaseous breath samples from a Tedlar bag through the ATM-coated microreactor (Fig. S1). After the exhaled breath sample had been pulled through the microreactor and evacuated under vacuum, the microreactor was disconnected. Finally, the ATM-VOC adducts were eluted from the microreactor with 100 *μ*L methanol to afford ≥99% ATM-VOC recovery 35. The eluted solution was directly analyzed by Fourier transform-ion cyclotron resonance mass spectrometry (FT-ICR-MS). A known amount of deuterated acetone completely reacted with ATM (ATM-acetone-d6) in methanol was added to the eluent as an internal reference (IR). The concentrations of all carbonyl compounds in exhaled breath were determined by comparison of the relative abundance with that of added ATM-acetone-d6 IR.

Exhaled breath samples of healthy smoker and nonsmoker controls (*n* = 88) and patients with pulmonary nodules (*n* = 129) were analyzed and the concentrations of all carbonyl compounds were determined. The measurement and recording of VOC concentrations in breath samples of patients was performed without knowledge of the clinical or pathologic diagnosis of cancer or absence thereof. The analytical data were later compared to the clinical results to determine sensitivity and specificity of the measurements.

### FT-ICR-MS instrumentation

The eluent was analyzed by a hybrid linear ion trap–FT-ICR-MS (Finnigan LTQ FT; Thermo Electron, Bremen, Germany) equipped with a TriVersa NanoMate ion source (Advion BioSciences, Ithaca, NY) with an electrospray chip (nozzle inner diameter 5.5 *μ*m). The TriVersa NanoMate was operated in positive ion mode by applying 2.0 kV with no head pressure. Initially, low-resolution MS scans were acquired over 1 min to ensure the stability of ionization, after which high mass accuracy data were collected using the FT-ICR analyzer. FT-MS scans were acquired for 8.5 min at a target mass resolution of 100,000 at 800 *m*/*z*. The AGC (automatic gain control) maximum ion time was set to 500 msec (but typically utilized <10 msec) and five “*μ*scans” were acquired for each saved spectrum; thus, the cycle time for each transformed and saved spectrum was about 10 sec. FT-ICR mass spectra were exported as exact mass lists into a spreadsheet file using QualBrowser 2.0 (Thermo Electron), typically exporting all the observed peaks. ATM and ATM-VOC adducts were assigned based on their accurate mass by first applying a small (typically <0.0005) linear correction based on the observed mass of the internal standard 35.

### Statistical data analysis

The measured carbonyl VOC concentrations in exhaled breath samples were separated into four groups: healthy controls (HC), non–small cell lung cancer (NSCLC), SCLC, and patients with benign pulmonary nodules (BN). The NSCLC group was further separated into adenocarcinoma and squamous cell carcinoma subgroups. All measured carbonyl VOC concentrations were analyzed by the Wilcoxon test to determine statistically significant differences between two groups. The Wilcoxon tests were performed using Minitab version 16.0.

## Results and Discussion

The efficiencies of carbonyl capture by the ATM-coated microreactor were characterized first by using single carbonyl standards and mixtures of carbonyl standards 34,35. The capture efficiencies are affected by the velocity of the VOC mixture flowing through the microreactor as well as the molar ratio of ATM/carbonyl compound. Capture efficiencies greater than 98% have been achieved for trace ketones and aldehydes under the optimized microreactor microstructure and operation conditions.

Prior to exhaled breath analysis, the concentrations of carbonyl VOCs from laboratory air, clinic room air, and street air samples were determined. Then, the concentrations of carbonyl VOCs in exhaled breath samples from 88 HC (45 smokers and 43 nonsmokers) and 129 patients with pulmonary nodules were measured. Carbonyl VOCs from C_1_ (formaldehyde) to C_12_ in the exhaled breath samples of the healthy subjects and the patients with pulmonary nodules have been detected. Of all carbonyl compounds, only formaldehyde, acetaldehyde, and acetone had concentrations slightly higher in exhaled breath than in environmental air. All other carbonyl compounds had at least 10 times higher concentration in exhaled breath than in air and some were not detected in air.

Diagnosis of the 129 patients with pulmonary nodules was made by either biopsy or resection in 124 patients. A pathologic diagnosis of lung cancer was confirmed in 97 patients, and benign nodules in 27 patients. The other five patients were clinically diagnosed with benign pulmonary nodules based on the shrinkage of nodule size for at least 6 months after the collection of breath samples. The 97 lung cancer patients were comprised of 9 with SCLC and 88 with NSCLC. Of the NSCLC patients, 33 were diagnosed with adenocarcinoma and 32 with squamous cell carcinoma. The remaining NSCLC patients (*n* = 23) were diagnosed either with poorly differentiated cancer or a mixture of cancer cell types.

**Figure 2:**
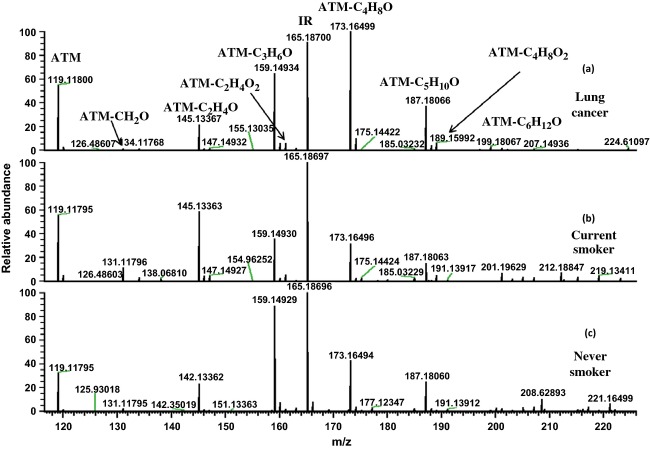
Fourier transform-ion cyclotron resonance mass spectrometry (FT-ICR-MS) spectra of breath samples. (a) Lung cancer patient, (b) smoker control, and (c) nonsmoker control. The peak of ATM is 2-(aminooxy)-*N*,*N*,*N*-trimethylethanammonium cations. The peak of IR is internal reference ATM-acetone-d6. All other peaks are ATM reacted with carbonyl VOCs. For example: ATM-C_2_H_4_O is ATM reacted with acetaldehyde and ATM-C_4_H_8_O is ATM reacted with 2-butanone.

The 2-butanone concentration (ATM-C_4_H_8_O in Fig. 2a) was typically the highest of all carbonyl VOCs in the exhaled breath of lung cancer patients. The acetaldehyde concentration (ATM-C_2_H_4_O in Fig. 2b) was the highest among healthy smokers, likely due to its abundance in cigarette smoke. Healthy nonsmokers typically had acetone (ATM-C_3_H_6_O in Fig. 2c) as the most concentrated carbonyl compound in their exhaled breath. Figure 2 shows that the lung cancer patient has a notably higher relative abundance of 2-butanone and 3-hydroxy-2-butanone (ATM-C_4_H_8_O_2_, Mw = 189.15982) (Fig. 2a) than the healthy smoker (Fig. 2b) and nonsmoker (Fig. 2c) in comparison with the abundance of IR (ATM-acetone-d6) (peak labeled as IR in Fig. 2). The Wilcoxon statistical test indicated that the concentrations of 2-butanone (*P *< 0.0001), 3-hydroxy-2-butanone (*P *< 0.0001), 2-hydroxyacetaldehye (*P *< 0.0001), and 4-hydroxyhexenal (4-HHE) (*P *< 0.0005) were significantly higher in the exhaled breath samples of the lung cancer patients compared to the HC group. The chemical structures of the identified VOCs of lung cancer in exhaled breath samples were confirmed by FT-ICR-MS/MS using 2-butanone, 3-hydroxy-2-butaone, and 2-hydroxyacetaldehyde purchased from Sigma-Aldrich, and 4-HHE from Cayman Inc. as standard references. The concentration ranges of these four VOCs for the group of the HC, the group of the patients with lung cancer (LC), and the group of the patients with BN are presented in Table 1. There are overlaps for the concentration ranges of these four VOCs for the studied groups. 2-butanone and 3-hydroxy-2-butanone have been recently reported as lung cancer markers in exhaled breath by Bajtarevic et al. 18 and Song et al. 13. However, there are no determined concentration ranges of these two compounds for diagnosis of lung cancer. 2-Butanone, 3-hydroxy-2-butanone, and 2-hydroxyacetaldehyde are present in ambient air. However, the concentrations of these VOCs in air were at least 10 times lower than in exhaled breath samples. In addition, 4-HHE was not detected in air. We thus conclude that the effects of environmental air on the concentrations of these four carbonyl VOCs in exhaled breath samples can be neglected. Therefore, we surmise that these carbonyl species are primarily from alveolar breath and their concentrations increase with cancer presence.

**Table 1:** The concentration ranges of the four carbonyl VOCs related to lung cancer.

VOCs (nmol/L)	HC (*n* = 88)	BN patients (*n* = 32)	LC patients (*n* = 97)	P* value
2-Butanone	0.45–2.34	0.79–4.25	1.78–8.38	<0.0001
3-Hydroxy-2-butanone	0.02–0.15	0.01–0.42	0.13–0.77	<0.0001
2-Hydroxyacetaldehyde	0.03–0.45	0.01–0.80	0.23–1.13	<0.0001
4-HHE	0.00007–0.009	0.00028–0.029	0.005–0.05	<0.0005

* All *P* values are between the group of healthy controls (HC) and the group of lung cancer (LC) patients. VOCs, volatile organic compounds; BN, patients with benign pulmonary nodules; 4-HHE, 4-hydroxyhexenal.

Table2 lists the total number of the four carbonyl VOCs in the concentration ranges of lung cancer as shown in Table 1 for the 129 patients with pulmonary nodules. All patients (*n* = 29) exhaling the four carbonyl VOCs at concentrations indicative of lung cancer were diagnosed with lung cancer, while 34 of 35 patients exhaling three carbonyl VOCs at concentrations indicative of lung cancer were diagnosed with lung cancer. There were two cancer patients without any of the four carbonyl VOCs at concentrations indicative of lung cancer. By defining a simple and practical diagnostic rule of an elevation in at least two of the four carbonyl VOCs as indicative of lung cancer, a sensitivity of 89.8% (87 correct prediction of a total 97 cancer patients) and a specificity of 81.3% (26 correct prediction of the total 32 benign pulmonary nodule patients) were obtained. Although these results are very promising for clinical application of diagnosis of lung cancer for patients with pulmonary nodules, there is a need for testing a much larger number of patients with pulmonary nodules in order to develop a reliable method for diagnosis of lung cancer.

**Table 2:** Total number of the carbonyl VOCs at concentration in the ranges of lung cancer shown in Table 1 in the breath samples of lung cancer (LC) and benign pulmonary nodule (BN) patients.

No. of VOCs	4	3	2	1	0
No. of LC patients	29	44	17	5	2
No. of BN patients	0	1	5	8	18

**Figure 3:**
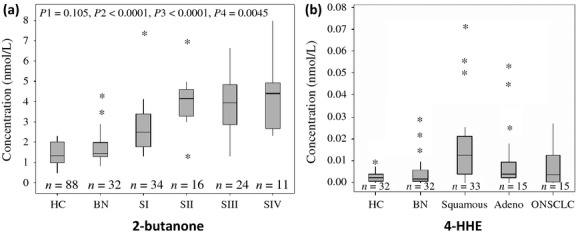
The relationship between carbonyl compounds and non–small cell lung cancer (NSCLC) stage and cancer type. (a) The relationship between the concentration of 2-butanone in exhaled breath and the cancer stages of NSCLC. HC, healthy controls; BN, patients with benign pulmonary nodules; SI to SIV, patients with stage I to stage IV NSCLC. The box plot presents the median, lower, and upper quartiles (25th–75th percentiles). p1 is the *P* value between HC and BN groups, p2 is the *P* value between HC and NSCLC, p3 is the *P* value between BN and NSCLC, and p4 is the *P* value between early-stage NSCLC (I and II) and advanced NSCLC (III and IV); (b) The concentration difference in 4-HHE among HC, BN, patients squamous cell carcinomas, and patients with poorly differentiated NSCLC or a combination of two types NSCLC (labeled as ONSCLC). The box plot presents the median, lower, and upper quartiles (25th and 75th percentiles). The *P* value between squamous cell carcinoma group and adenocarcinoma group is 0.03, and the *P* value between squamous cell carcinoma group and ONSCLC group is 0.066.

To determine whether the carbonyl VOC markers could be related to lung cancer stages, the concentrations of 2-butanone, 3-hydroxy-2-butanone, 2-hydroxyacetaldehye, and 4-HHE in 34 patients with stage I, 16 patients with stage II, 24 patients with stage III, and 11 patients with stage IV of NSCLC were also analyzed by the Wilcoxon test. The concentrations of 3-hydroxy-2-butanone, 2-hydroxyacetaldehye, and 4-HHE are not related to lung cancer stages. Figure 3(a) shows that the concentration of 2-butanone can be related to stage I lung cancer. The concentration of 2-butanone in the exhaled breath samples of patients with stage I lung cancer was significantly higher than that in the HC and the patients with benign pulmonary nodules, but significantly lower than that in the patients with stages II to IV lung cancer. There is no significant difference in 2-butanone concentrations in patients with stages II through IV lung cancer.

To determine the relationship of these carbonyl VOCs to the cancer histology of NSCLC, we analyzed the concentrations of the four VOC markers in 33 patients with adenocarcinomas, 32 patients with squamous cell carcinomas, and 15 patients with either poorly differentiated NSCLC or a combination of two types of NSCLC (labeled as ONSCLC in Fig. 3b). The patients with squamous cell carcinomas have significantly higher concentrations of 4-HHE than the patients with adenocarcinomas (*P* = 0.03) (Fig. 3b). However, there is no significant difference in the concentrations of 4-HHE between the group of adenocarcinomas and ONSCLC group. There is no significant difference in the concentrations of 4-HHE in the different stages of either adenocarcinoma or squamous cell carcinoma patients (Fig. S2).

**Figure 4:**
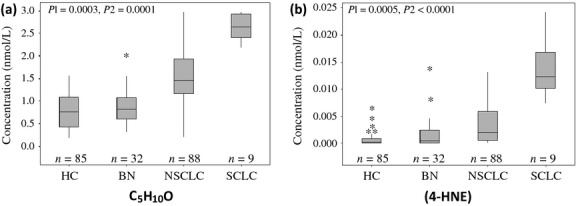
Carbonyl compounds for identification of SCLC and NSCLC. (a) The box plot of concentrations of C5H10O in the exhaled breath samples of the healthy controls (HC), and the patients with BN, SCLC, and NSCLC. The box plot presents the median, lower, and upper quartiles (25th and 75th percentiles). p1 is the *P* value between HC and lung cancer patients (LC); p2 is the *P* value between NSCLC and SCLC. (b) The box plot of concentration of 4-hydroxynonenal (4-HNE) in exhaled breath of HC, patients with BN, SCLC, and NSCLC. p1 is the *P* value between HC and lung cancer patients (LC); p2 is the *P* value between NSCLC and SCLC patients.

In order to develop breath analysis results for future diagnosis of patients with SCLC, the concentrations of all carbonyl VOCs in breath samples of five patients with limited-stage SCLC and four patients with advanced-stage SCLC were analyzed and compared with the patients with NSCLC(*n* = 88). There was no significant difference in the concentration ranges of 2-butanone, 3-hydroxy-2-butanone, 2-hydroxyacetaldehye, and 4-HHE in the SCLC patients when compared to the NSCLC patient group (Fig. S3). However, Figure 4 shows that there is a significant increase in the concentrations of 4-hydroxynonenal (4-HNE) (*P* < 0.0001) and C_5_H_10_O (*P* = 0.0001) for the SCLC patients. We have used GC-MS to determine that C_5_H_10_O in exhaled breath was a mixture of pentanone and *n*-pentanal. Pentanal, hexanal, octanal, and nonanal in exhaled breath were reported to have significantly higher concentrations in exhaled samples of lung cancer patients (*n* = 12) than in that of health controls (*n* = 24) 23. However, this work found that pentanal was significantly higher only in exhaled breath of SCLC patients and did not find significantly higher concentrations of hexanal, octanal, and nonanal in exhaled breath of lung cancer patients. To the best of our knowledge, the results of significantly higher concentrations of 4-HHE and 4-HNE, and 2-hydroxyacetaldehyde in the exhaled breath of NSCLC and SCLC patients are for the first time reported. Recent experimental results have pointed to an inflammatory origin as a possible trigger of lung cancer 37,38. Both 4-HHE and 4-HNE are well known as products of lipid peroxidation caused by reactive oxygen species that have been attributed to dysregulations of lung cancer 32,33.

## Conclusion

In conclusion, using a silicon microreactor coated with ATM, four carbonyl VOCs in exhaled breath have been identified that when at elevated concentrations reliably diagnose lung cancer. Specifically, the concentrations of 2-butanone, 3-hydroxy-2-butanone, 2-hydroxyacetaldehye, and 4-HHE in breath are readily determined by FT-ICR-MS analysis of the respective ATM-VOC adducts, and elevated concentrations of these adducts relative to concentrations in healthy patients, or even patients with benign pulmonary nodules, indicate the presence of lung cancer. The concentration of 2-butanone can be used to distinguish stage I lung cancer from stages II through IV. Furthermore, the concentration of 4-HHE may be used to distinguish squamous cell carcinoma from adenocarcinoma and other NSCLC, and the concentrations of 4-HNE and C_5_H_10_O can be used to distinguish SCLC patients from NSCLC patients. These findings have immediate application as an accurate, noninvasive means for the diagnosis of lung cancer. Further study may show that they are an effective means of early detection of lung cancer in conjunction with CT scanning and in monitoring for the recurrence of lung cancer postresection.

## Supporting Information

Figure S1 Schematic setup for the capture of carbonyl VOCs from air and gaseous breath samples collected in the Tedlar bags.

Figure S2 The box plots the concentration of 4-HHE in exhaled breath samples of nonsmoker controls (NS), smoker controls (SC), and the patients with stage I (SSI) to stage IV (SSIV) of NSCLC.

Figure S3 The box plots the concentration of (A) 2-butanone, (B) 3-hydroxy-2-butanone, (C) 2-hydroxyacetaldehyde, (D) 4-HHE in exhaled breath samples of nonsmoker controls (NS), smoker controls (S), and the patients with NSCLC and SCLC.
