# Supplementary figures and images for: Noninvasive detection of lung cancer using exhaled breath
Source: Cancer Med. 2013 Nov 20;3(1):174–81. doi: 10.1002/cam4.162 (PMC3930402; doi:10.1002/cam4.162)

**Fig. S1**

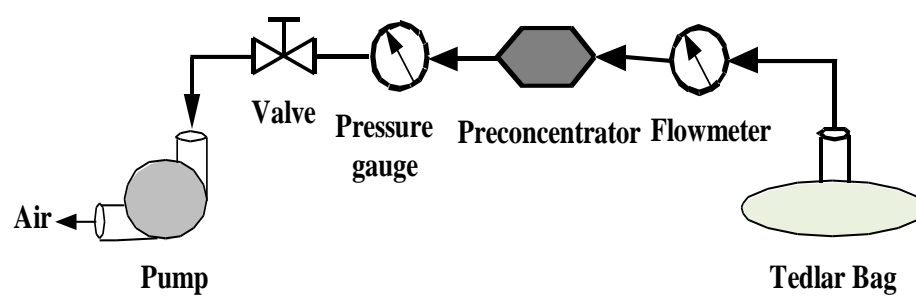

Supplement: Figure S1 — Schematic setup for the capture of carbonyl VOCs from air and gaseous breath samples collected in the Tedlar bags. [file cam40003-0174-sd1.pdf]

**Fig. S2**

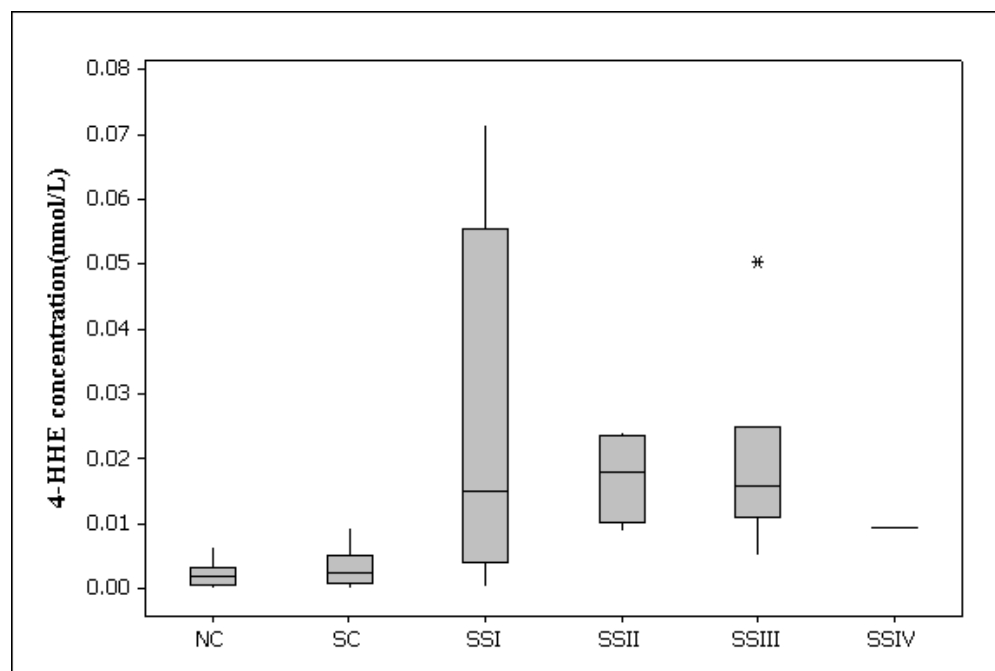

Supplement: Figure S2 — The box plots the concentration of 4-HHE in exhaled breath samples of nonsmoker controls (NS), smoker controls (SC), and the patients with stage I (SSI) to stage IV (SSIV) of NSCLC. [file cam40003-0174-sd2.pdf]

**Fig. S3**

**(a) 2-Butanone**

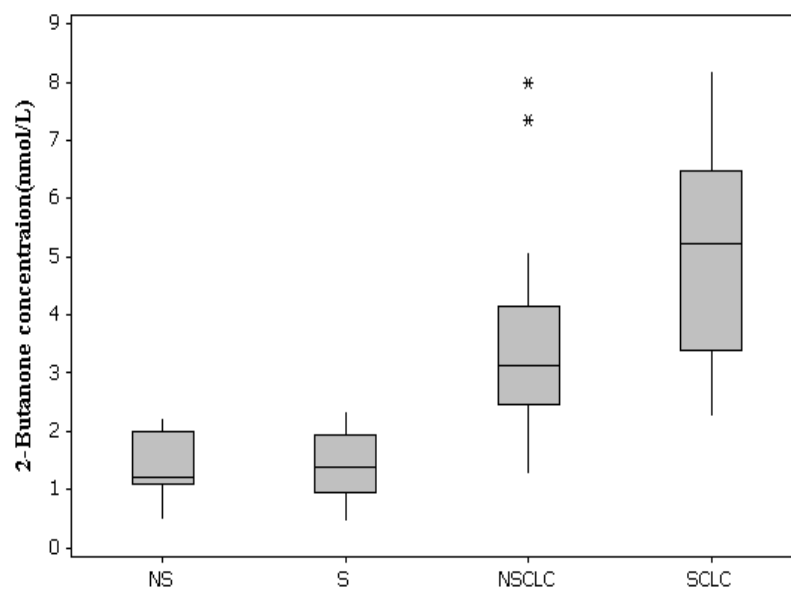

**(b) 3-hydroxy-2-butanone**

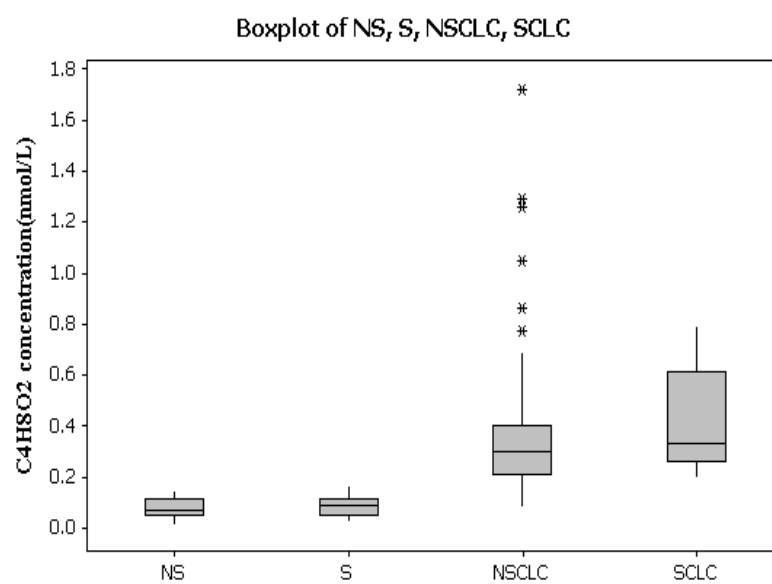

**(c) 2-hydroxyacetaldehyde**

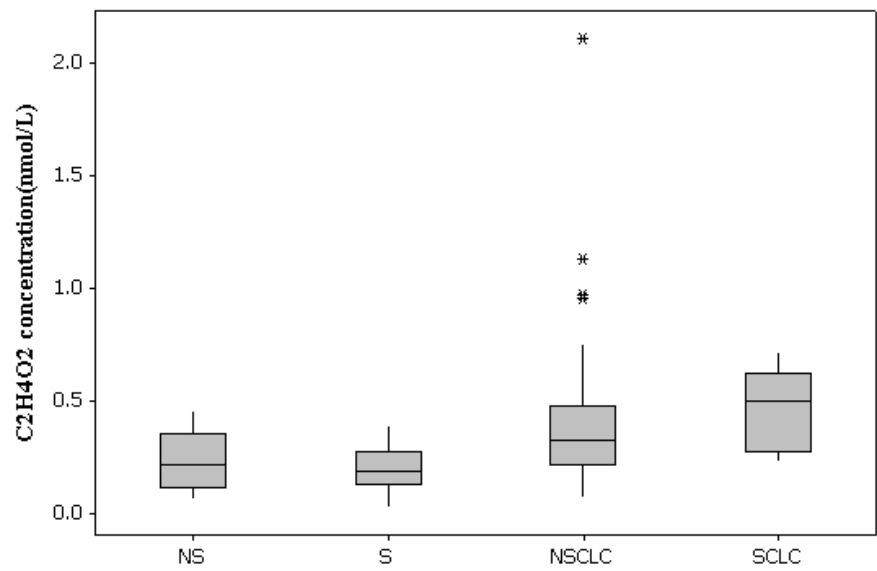

**(d) 4-HHE**

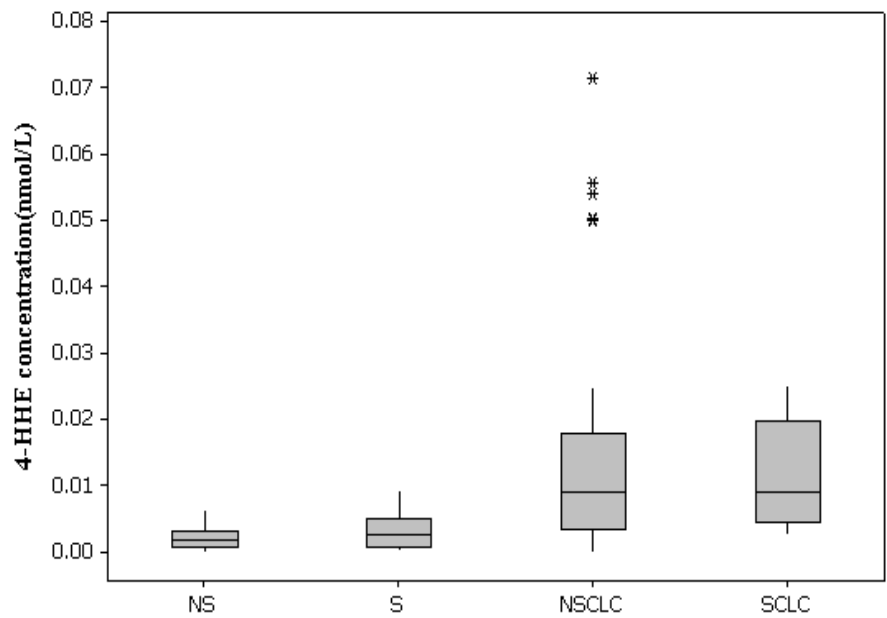

Supplement: Figure S3 — The box plots the concentration of (A) 2-butanone, (B) 3-hydroxy-2-butanone, (C) 2-hydroxyacetaldehyde, (D) 4-HHE in exhaled breath samples of nonsmoker controls (NS), smoker controls (S), and the patients with NSCLC and SCLC. [file cam40003-0174-sd3.pdf]
